# Rare earth element geochemistry of outcrop and core samples from the Marcellus Shale

**DOI:** 10.1186/s12932-015-0022-4

**Published:** 2015-06-26

**Authors:** Clinton W Noack, Jinesh C Jain, John Stegmeier, J Alexandra Hakala, Athanasios K Karamalidis

**Affiliations:** Department of Civil and Environmental Engineering, Carnegie Mellon University, PH 118L, Pittsburgh, PA 15213 USA; URS, Washington Division, National Energy Technology Laboratory, Pittsburgh, PA 15236 USA; Center for Environmental Implications of Nanotechnology (CEINT), Durham, USA; National Energy Technology Laboratory, Pittsburgh, PA 15236 USA

**Keywords:** Marcellus Shale, Rare earth elements, Material characterization, ICP-MS, X-ray diffraction, Brine management, Waste disposal

## Abstract

In this work, the geochemistry of the rare earth elements (REE) was studied in eleven outcrop samples and six, depth-interval samples of a core from the Marcellus Shale. The REE are classically applied analytes for investigating depositional environments and inferring geochemical processes, making them of interest as potential, naturally occurring indicators of fluid sources as well as indicators of geochemical processes in solid waste disposal. However, little is known of the REE occurrence in the Marcellus Shale or its produced waters, and this study represents one of the first, thorough characterizations of the REE in the Marcellus Shale. In these samples, the abundance of REE and the fractionation of REE profiles were correlated with different mineral components of the shale. Namely, samples with a larger clay component were inferred to have higher absolute concentrations of REE but have less distinctive patterns. Conversely, samples with larger carbonate fractions exhibited a greater degree of fractionation, albeit with lower total abundance. Further study is necessary to determine release mechanisms, as well as REE fate-and-transport, however these results have implications for future brine and solid waste management applications.

**Electronic supplementary material:**

The online version of this article (doi:10.1186/s12932-015-0022-4) contains supplementary material, which is available to authorized users.

## Background

Unconventional natural gas and oil resources include tight-gas sands, coal bed methane, and organic-rich black shales [1]. One such shale is the middle-Devonian Marcellus, a ubiquitous formation throughout much of the Appalachian Basin [2]. Saline, metal-enriched produced waters from the Marcellus [3] are an environmental concern for their potential to contaminate shallow groundwater or surface water [4]. While the water–rock interactions that govern the dissolved constituents of produced waters are not well understood [5], information regarding the metal contents and mineralogy of the Marcellus Shale is necessary for assessing the potential for metal mobilization in situ or upon disposal of waste cuttings.

Beyond assessing and managing risk, thorough source-rock characterization can elucidate other applications of dissolved constituents in produced waters. Capable source identification tools are necessary in regions, such as the Appalachian basin, where multiple sources of salinity overlap [6]. For example, unique trace metal and isotope chemistry can be used as naturally occurring indicators, or fingerprints, of water–rock interactions and fluid migration and mixing [5–7]. The rare earth elements (REE) have been extensively studied in sedimentary formations (and geologic media in general), typically in the context of inferring depositional environments [8, 9] or diagenic processes [10–12]. Thus, the REE are potential fingerprints of water–rock interactions as well as geochemical signatures of brine sources [13, 14].

Despite years of interest in and study of the formation, [15–17] current data on the trace-metal lithogeochemistry of the Marcellus is limited. Chermak and Schreiber [18] provided a thorough compilation and analysis of published studies of various oil and gas shales, focusing on the pyrite/calcite balance of the mineralogy as well as the implications of trace-metal abundance for solid waste disposal. However, the majority of reported analyses within that study for the Marcellus were from three core samples: two from Bracht [19] and one from Werne et al. [20]. Additionally, little or no discussion exists regarding the REE profiles of the Marcellus Shale. Chiarenzelli et al. [21] included data from a depth-stratified core of the Marcellus in New York State for comparative purposes when using REE profiles when studying the Popple Hill Gneiss. However, the REE data are presented in summary, unaccompanied by mineralogical analysis.

Given this lack of prior art, the objective of this study was to expand the knowledge of REE occurrence in the Marcellus Shale through investigations of elemental abundance and mineralogy. This objective was addressed with three tasks: (1) determine the REE abundance in samples of the Marcellus Shale by LiBO_2_ fusion and ICP-MS analysis, (2) study the mineralogy of these samples using X-ray diffraction, and (3) hypothesize mineralogy of the REE via statistical analysis of experimental results. Where appropriate, comparisons were made between sample types (i.e. core and outcrop) and between outcrop localities (i.e. northern or southern). This data can subsequently be used to inform focused studies of the potential for metal release by various mechanisms.

## Results and discussion

### Sample acquisition

**Figure 1:**
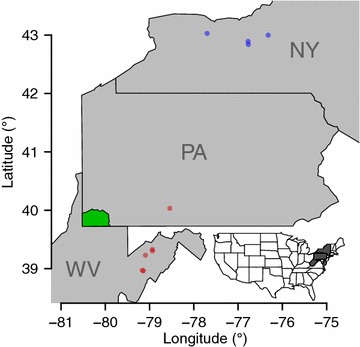
Approximate locations of outcrop (*blue* and *red circles*) and core (in Greene County, PA; *colored green*) samples. Outcrops were collected as fresh exposures.

Fresh exposures from Marcellus Shale outcrops (N = 11) were collected as part of research at the Department of Energy, National Energy Technology Laboratory (NETL) in the Industrial Carbon Management Initiative (ICMI) between May 2010 and September 2011. Four outcrops were sampled from northern, surface exposures in New York State (NY) while the remaining outcrop samples originated from southern, surface exposures in West Virginia (WV) and Pennsylvania (PA). Outcrop samples were primarily from the Union Springs or Oatka Creek members of the Marcellus Shale. Samples (N = 6) at six depth intervals (between 7,780 and 7,920 ft below ground surface) from a single core were provided by an industrial partner, under terms of confidentiality, operating in Greene County, PA. Locations of core and outcrop samples can be seen in Figure 1. Details of the outcrop samples, including lithologic and stratigraphic descriptions, are given in Additional file 1: Table S1.

While the samples studied here were not exclusively from gas-bearing members of the Marcellus, if the REE are to be used as tracers it is important to have thorough characterization of the REE in over- and underlying strata. Induced fractures (and therefore fluids) often propagate “out-of-zone” and, at times, hundreds of meters vertically above and below the perforation midpoint [22]. Therefore the variety of strata (within the Marcellus) studied here are generally of interest for naturally occurring tracer applications.

### Rare earth element abundance, correlations, and profiles

**Figure 2:**
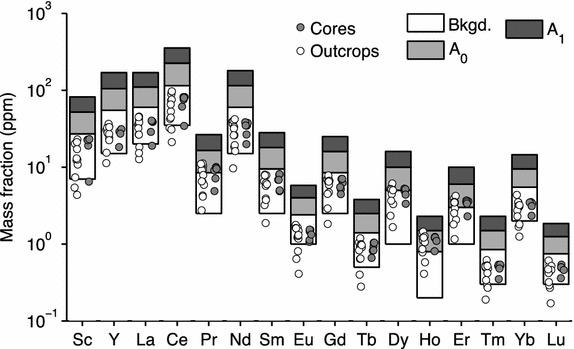
Rare earth element abundance in Marcellus Shale outcrop (*open symbols*; N = 12 for all analytes) and core samples (*closed symbols*; N = 6). Geochemical background (Bkgd.), anomalous (A_0_), strongly anomalous (A_1_) mass fraction ranges of world black-shales according to Ketris and Yudovich [10].

Concentrations of the study analytes are summarized in Figure 2, with sample-wise results presented in Table 1. In general, the REE varied over three orders of magnitude with 95% of all measurements in the range between 0.324 and 75.2 ppm. As expected, the REE exhibited a “zig-zag” pattern of abundance, consistent with the Oddo-Harkins effect [23]. Several samples were enriched—relative to world black shales [24]—in Pr, Dy, Ho, and Er, however most samples fell within the typical range for black shales compiled by Ketris and Yudovich [24].

**Table 1:** Sample-wise results of LiBO_2_ fusion and ICP-MS analysis of Marcellus Shale samples

Sample name	Sc	Y	La	Ce	Pr	Nd	Sm	Eu	Gd	Tb	Dy	Ho	Er	Tm	Yb	Lu
Bedford, PA	7.37	22.5	15.3	30.8	4.14	16.2	3.74	0.81	4.50	0.65	3.67	0.86	2.53	0.34	2.3	0.32
Canoga, NY (OCM)^a^	13.0	33.2	26.4	44.2	6.54	26.1	5.99	1.31	6.49	0.97	5.02	1.21	3.48	0.52	3.22	0.47
Canoga, NY (OCM)^a^	12.3	33.6	26.5	42.5	6.44	26.1	5.71	1.22	6.96	0.93	4.83	1.20	3.47	0.48	3.04	0.48
1-DGLS	6.51	29.5	19.0	34.4	4.89	20.0	4.88	1.07	5.16	0.82	4.77	1.20	3.51	0.52	3.52	0.54
Petersburg, WV (N)	12.6	27.0	32.2	65.4	7.90	31.8	6.23	1.27	5.89	0.83	4.47	1.06	2.90	0.42	2.73	0.42
C-7789	19.1	18.3	28.7	61.2	7.14	26.6	4.99	1.13	4.46	0.66	3.34	0.81	2.30	0.35	2.33	0.36
Whip Gap, WV	5.42	11.3	12.7	21.1	2.75	9.65	1.88	0.41	1.83	0.28	1.66	0.41	1.16	0.19	1.25	0.17
Burlington, WV (1)	21.2	27.8	44.7	97.0	11.0	42.2	7.83	1.60	6.38	0.91	4.95	1.11	3.39	0.51	3.12	0.46
Canoga, NY (USM)	17.2	32.6	38.4	75.6	9.17	33.4	6.55	1.48	6.62	0.97	5.08	1.20	3.51	0.49	3.15	0.44
C-7838	22.7	30.5	38.6	75.9	9.08	34.8	6.82	1.46	6.25	0.93	4.93	1.20	3.60	0.52	3.32	0.50
Petersburg, WV (W)	23.0	30.6	45.5	96.5	11.2	40.3	7.86	1.62	6.62	1.00	5.23	1.26	3.53	0.53	3.51	0.52
C-7907	10.8	15.4	37.1	53.0	5.90	19.3	3.21	0.64	2.90	0.40	2.34	0.58	1.81	0.27	1.81	0.29
C-7801^b^	22.7	29.2	39.6	81.6	10.0	37.5	7.95	1.53	7.01	1.04	5.25	1.24	3.69	0.54	3.39	0.49
C-7801^b^	22.5	31.3	39.5	81.9	9.88	38.4	8.14	1.54	7.05	1.04	5.17	1.22	3.54	0.54	3.47	0.50
Le Roy, NY	20.2	36.6	35.4	73.4	9.26	36.2	7.99	1.77	7.81	1.19	6.13	1.45	4.23	0.62	4.35	0.61
Marcellus, NY	20.9	33.6	42.5	88.7	10.3	39.3	7.80	1.64	7.19	1.05	5.51	1.33	3.91	0.53	3.49	0.54
Burlington, WV (2)	4.37	24.0	18.2	32.5	4.28	18.4	3.92	1.18	4.70	0.64	3.31	0.78	2.08	0.29	1.68	0.27
C-7813	23.1	27.3	40.4	79.5	9.52	34.8	6.82	1.33	5.51	0.83	4.4	1.10	3.37	0.48	3.12	0.46

All results are mass fractions, reported in parts per million (mg/kg) with three significant-digit precision. Average analytical uncertainty was 3.4% from 5 sweeps during analysis.

^a, b^Method duplicates.

The REE were also highly, positively correlated in these samples. The median interelement correlation (Spearman’s $\rho$) was 0.81 while 95% of all correlations fell between 0.47 and 0.98. The minimum observed correlation (0.33) was between La and Y. In general, REE tended to correlate most strongly with the nearest elements, with Sc correlating better with the LREE and Y with the HREE (Additional file 1: Figure S3). Overall, the high correlations exhibited in these samples were consistent with correlations determined in aqueous media [25], which was expected given the ubiquitous occurrence and coherent chemical properties of the REE.

Rare earth element concentrations were statistically compared between core and outcrop samples and between outcrop localities to determine if presumed weathering of outcrops or regional variations might yield systematically different REE concentrations. Despite the REE concentrations in the outcrop samples appearing to be more variable than in the core samples (e.g. Eu in Figure 2), no statistically significant differences in element variability were determined between the outcrop and core samples (Ansari-Bradley test for difference in scale parameter; $P \approx 1$ for all elements following Bonferroni–Holm corrections for multiple comparisons). Similarly, no statistically significant differences were found in the central tendencies of any of the REE between the two sample types (Wilcoxon rank-sum test for location shift; $P \approx 1$ for all elements, corrected for multiple comparisons). Analogous, parametric tests (Bartlett test for homogeneity of variance and a *t* test) were performed, also indicating no significant differences (Additional file 1, Section: “Outcrop-core statistical comparison”).

Testing of reduced dimension variables, such as the total REE content, similarly exhibited no differences between sample types. This could indicate that surface weathering processes did not appreciably alter the REE composition. Alternatively, the small sample size leads to aggregation of the samples as “outcrops” since insufficient samples were available to compare among members of the Marcellus (e.g. Union Springs vs. Oatka Creek). This could lead to false negative test results as inter-strata variability could obscure variability due to weathering.

Application of the PERMANOVA test further confirmed the lack of difference between the two sample types in bulk REE content ($P > 0.5$ from 10,000 permutations). While the apparent differences in dispersion or variance between the types may not be detectable given the small sample size, the similarity of medians corresponds with the findings of Chermak and Schreiber [18], where numerous, non-REE analytes agreed between core samples from different geographies within the Marcellus.

Similar results (i.e. no statistically significant differences) were obtained for uni- and multivariate comparisons between northern and southern outcrop samples. This indicates that inter-regional variability of the bulk REE composition of the shale may be less significant than intra-regional variability (i.e. at the stratigraphic or mineralogical scale). However, the current dataset is insufficient to make meaningful, statistical comparisons between stratigraphic groups.

**Figure 3:**
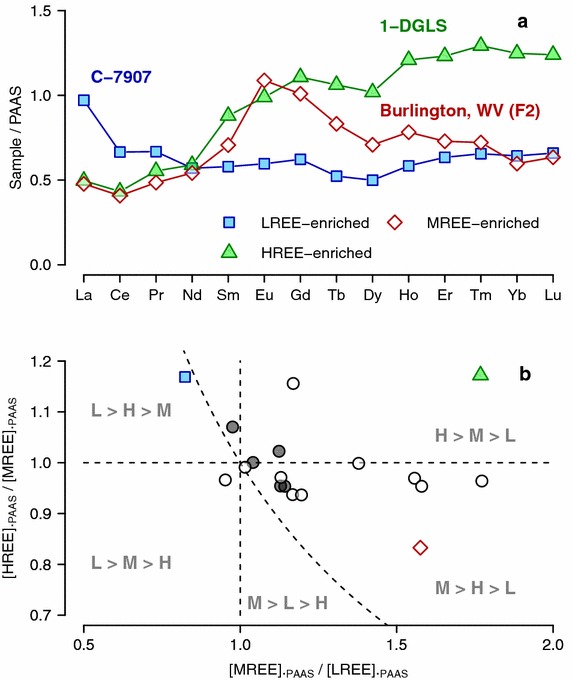
Post-Archaean Average Shale (PAAS) normalized REE profiles for Marcellus Shale outcrop and core samples. **a** Representative REE profiles of samples from this study exhibiting HREE-, MREE-, and LREE-enrichment. **b** Averaged, element ratio biplot. Points summarize the averaged, PAAS-normalized, interelement ratios for each sample. The *square*, *diamond*, and *triangle* in (**b**) correspond to the REE profiles plotted with matching markers in (**a**) while *circles* represent all remaining samples. *Filled symbols* (N = 7) indicate core samples, while *open symbols* (N = 11) indicate outcrops. The generic order of normalized REE weight ranges (HREE, MREE, LREE) are given in *grey* (e.g. “H > M>L” in the *upper right portion* of the plot); see Stolpe et al. [35] and Noack et al. [25] for further interpretation.

REE profiles of these samples were variable, with enrichments of all REE weight classes—LREE, MREE, and HREE—observed in PAAS-normalized patterns (Figure 3a, b). However, most samples exhibited LREE depletion (that is they had MREE/LREE and HREE/LREE ratios >1) with MREE enrichments predominating (Figure 3b). Similarly, some samples exhibited negative Ce anomalies (Ce* < 1), but most samples had Ce and Eu anomalies near 1 (anomalies not pictured) fitting with an anoxic to sulfidic, sedimentary environments such as those proposed for the Marcellus Shale [16, 20, 26]. No statistically significant differences were observed in REE patterns as either sample type (core vs. outcrop) or sample locality (North vs. South). Taken together, these results imply that variability in the REE profiles of the Marcellus Shale is dominated at the mineral scale.

### Crystalline mineralogy determined by XRD

**Figure 4:**
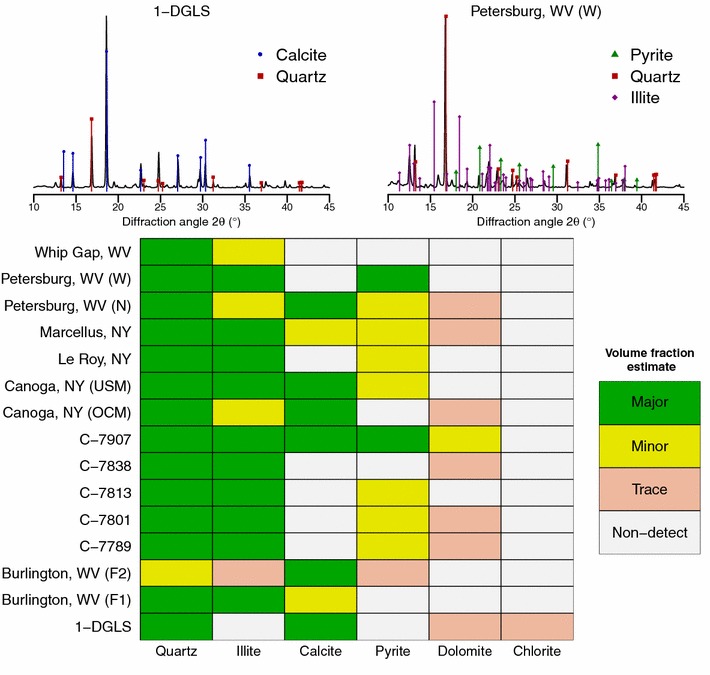
Summary of semi-quantitative XRD analysis of samples in this study. Outcrop samples are denoted by their location and core samples as “C-depth below ground surface”. Sample “1-DGLS” is a core sample, but was collected from an unspecified depth. Additional information regarding these samples is found in Additional file 1: Table S1. (*Top*) Example diffraction patterns with major matched peaks for two samples. (*Bottom*) Heat map of semi-quantitative XRD analyses for samples in this study. Model spectra are referenced in Additional file 1: Table S4.

The results of semi-quantitative XRD analyses are presented in Figure 4. The predominant crystalline mineral phases identified in these samples were quartz (classified as a major phase in 14 of 15 samples analyzed and as minor in 1 of 15), illite (10/15 major, 3/15 minor, 1/15 trace, and 1/15 non-detect), pyrite (2/15 major, 7/15 minor, 1/15 trace, and 5/15 non-detect), and calcite (6/15 major, 2/15 minor, and 7/15 non-detect). These results agree with the compilation of Chermak and Schreiber [18], who found other Marcellus samples to be predominantly phyllo- and tecto-silicates, while other gas shales (such as the Antrim and Eagle Ford) were more carbonaceous.

**Figure 5:**
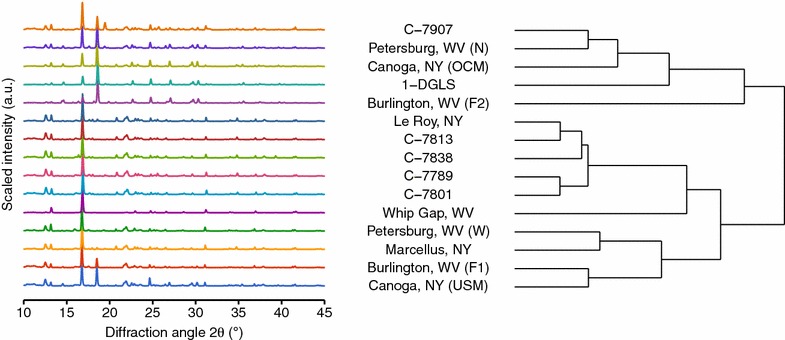
Comparison of X-ray diffraction patterns for samples in this study. Outcrop samples are denoted by their location and core samples as “C-depth below ground surface”. Sample “1-DGLS” is a core sample, but was collected from an unspecified depth. Additional information regarding these samples is found in Additional file 1: Table S1. (*Left*) Background-subtracted XRD spectra for samples in this study. Intensity was vertically scaled to display all samples simultaneously. (*Right*) Cluster dendrogram of XRD patterns. Clusters and linkage heights calculated via an average, unweighted distance algorithm. Intersample distances calculated as one minus the inter-sample Spearman’s $\rho$ correlation coefficient over the $2\theta$ range of 10°–45°.

Comparisons among diffraction spectra and hierarchical cluster analysis of these spectra for all samples are found in Figure 5. The cluster analysis shows some potential differences between core and outcrop samples as four of six core samples cluster strongly (along with one outcrop). However, the PERMANOVA test indicates no statistically significant differences between the XRD patterns of either cores or outcrops (P > 0.1 from 10,000 permutations). If the two disparate core samples, “1-DGLS” and “C-7907” are removed from the analysis, a slightly significant PERMANOVA result is achieved (P < 0.05 from 10,000 permutations). The primary mineralogical difference between these two core samples and the other cores is the inferred presence of a major calcite phase (Figure 4). These samples (“1-DGLS” and “C-7907”) also exhibited the greatest REE profile fractionation: in Figure 3a, b these samples are the green triangle and the blue square, respectively, which exhibit significant profile fractionation.

Similarly, the results of cluster analysis provide little confidence in discernable, mineralogical differences between the regionalized outcrop samples. PERMANOVA testing confirms this observation, with no significant differences as a function of location (P > 0.5 from 10,000 permutations). However, the apparent lack of regionality (with respect to these mineralogical and elemental analyses) may be an artifact of sample size (as other geochemical parameters are known to be highly, regionally variable in the Marcellus play [5, 7]) or may arise from the pooling of samples from unique strata.

### Relationships between REE profiles and mineralogy

The Mantel test was used to test for correlation between intersample distances calculated as a function of REE abundance and XRD spectra correlations. A moderate, positive correlation was observed (Spearman’s $\rho$ = 0.53, P < 0.001), indicating that differences in the crystalline mineralogy of the samples is a significant control on REE profile variability. This hypothesis was further explored by applying Wilcoxon tests to both the degree of fractionation metric and the total REE content using the semi-quantitative XRD results for each mineral as the predictor variable.

**Figure 6:**
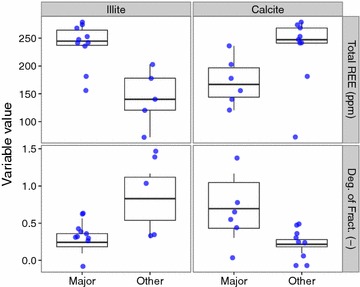
Distribution of total REE and degree of fractionation in samples with and without major fractions of illite or calcite. Distributions are depicted as standard *boxplots* [41], where the *thick*, *black line* depicts the median; the *boxed range* represents the 25th–75th percentile, or inter-quartile range (IQR); and the *thin whiskers* denote all measurements within 1.5 times the IQR above or below. Individual values of all observations are shown as *blue dots*. Statistically significant differences were found via the Wilcoxon rank sum test (H_0_: no difference in medians, P < 0.05) between groups for both variables and both minerals, as discussed in the text.

In this analysis, the presence of major illite or calcite phases was shown to have significant, contrasting effects on the REE abundance and fractionation (Figure 6). Total REE abundance showed a strong positive correlation with illite-enriched samples (P < 0.005). The Hodges–Lehmann estimator (HL) indicates that samples with a major illite phase had approximately 98 ppm more total REE (95% CI: 39–158 ppm) than samples without a major illite phase. Additionally, samples with major illite phases were between 14 and 112% less fractionated than samples without a major illite phase (HL 95% CI; P < 0.01). The latter finding seems intuitive as the degree of fractionation is calculated relative to a composite of clayey shales (PAAS). This result also indicated that the bulk of the REE concentration is likely found in the illite (or other clay) phases of the samples or in trace phases correlated with the clays. The mechanism of REE occurrence (i.e. sorbed or structurally incorporated) in these phases is not immediately elucidated, as the separate mineralogical fractions of the shale were not directly analyzed. Abanda and Hannigan [27] found that approximately 70% of the total REE content was likely associated with the silicate/clay fraction of Utica shale samples.

Conversely, samples with more calcite were between 54 and 400% more fractionated (HL 95% CI; P < 0.005), with 6–120 ppm less total REE than samples without a major calcite phase (HL 95% CI; P < 0.05), corroborating the conclusions drawn regarding differences in core samples, where dissimilar samples had a significant calcite fraction (Figures 3, 4). LREE-depletion has been observed in carbonate fractions of shales, potentially being excluded from the crystal lattice while MREE and HREE, with more similar ionic radii to Ca, are coprecipitated [27].

These postulates are supported by analyzing correlations between the major elements of the shale (i.e. Al, Ca, Fe, K, Mg, Na, and Si; reported for the outcrop samples studied here by Dilmore et al. [28]) and the total REE content as well as the degree of REE-profile fractionation (Additional file 1: Figures S4, S5). Namely, strong positive correlations were observed between total REE content and Al, Fe, K, Mg, and Na. This supports the hypothesis of total REE correlating with clay phases. Given the general abundance of these elements in all geologic media, substantial conclusions cannot be drawn on this data alone. However, Ver Straeten et al. [17] utilized related multivariate statistics to infer mineral inputs into the Devonian Appalachian Basin. Similar to Condie [29], no correlation was observed between total REE and P, indicating that minor phosphate minerals, which can be strong REE accumulators [30, 31], did not contribute significantly to the REE content of these samples.

The implications of these hypothesized mineral associations can be related to the potential for these shales to release REE during hydraulic fracturing. Since the REE may be structurally bound within the clays (as opposed to sorbed at surface sites) [32], it is possible that produced water REE profiles will not resemble those of the bulk shale. Yan et al. [33] found the REE to reside predominantly in the fine-grained fraction of a glacial till, clayey aquitard, but associated evenly between seven mineral fractions (elucidated through sequential leaching); the REE profiles of the adsorbed and exchangeable cations fraction, which were MREE- to HREE-enriched accounting for 9–10% of the total REE in those samples [33], most closely resembled the majority of profiles observed here. Conversely, the more readily soluble fractions (such as the carbonates, which often produce LREE-depletion [27]) may be responsible for REE profiles observed in produced waters, which could be used for source identification in the event of brine intrusion or waste spillage. More study is necessary to determine the release mechanisms of the REE under conditions relevant to hydraulic fracturing and solid waste disposal.

## Conclusions

Understanding trace metal geochemistry in shales and hypersaline brines is necessary in the face of expanding global development of unconventional oil and gas reserves through horizontal drilling and high-volume, hydraulic fracturing. Characterizing and managing the risk of fresh water contamination by solid and liquid wastes associated with these developments starts with an understanding of the geochemistry of compounds of interest in the host shales.

Stimulation of these shales during hydraulic fracturing will modify natural rates, extents, and pathways of weathering. These analyses can serve as a starting point for further investigation into the risk of metal mobilization during hydraulic fracturing, solid waste disposal, and throughout the well lifetime. Additionally, these tests provide a basis for understanding the capabilities for leached elements to serve as tracers of water–rock interactions.

## Experimental

### Materials

For sample fusion, lithium metaborate (LiBO_2_) was acquired from Acros Organics (99% purity; Lot # A0317552). Trace-metal grade nitric acid (HNO_3_) was used for fusion dissolution and as the background solvent for ICP-MS analysis (BDH ARISTAR^®^ Plus, VWR; assay 69 wt%; Lot # 1113050). Single element standard solutions (~1,000 µg/L) of the REE and all elements necessary for internal and external standardization were obtained from inorganic ventures. All acid dilutions were performed on a gravimetric basis using ultrapure water (ASTM Type I, 18.2 MΩ/cm), prepared using a Barnstead NANOpure^®^ water purification system.

### Rare earth element abundance analysis

Aliquots (~100 mg) of finely powdered sample were fused with ~1 g LiBO_2_ in graphite crucibles at 1,000°C for 30 min to yield a homogenous, molten fusion. Samples were quickly removed from the furnace and poured into pre-weighed 125 mL HDPE bottles partially filled with 5% HNO_3_. After all fusions were dispensed, the total volume of digestate was brought up to ~100 mL with 5% HNO_3_ and weighed. Bottles were then placed into an ultrasonic bath for 2 h to break apart any remaining particulate matter.

The resulting digestates were analyzed by inductively coupled plasma mass spectrometry (ICP-MS) for Sc, Y, and the lanthanides (herein collectively referred to as the REE). An internal and external standardization technique was used to correct for spectral interferences, matrix effects, and instrument drift during analysis [34]. All analyses were performed on an Agilent 7700x ICP-MS with He-mode octopole reaction cell; instrumental operating parameters are given in Additional file 1: Table S2. USGS certified reference materials (CRM) BCR-2 and SGR-1 were analyzed to assess method accuracy (Table 2). Confidence in the analytical results was gained by testing the central tendency and dispersion in CRM and method-duplicate errors; the relevant techniques and results are included in the Additional file 1 (Section: “Statistical validation of CRM and duplicate analyses”).

**Table 2:** Analytical method quality assurance for USGS reference materials BCR-2 and SGR-1 with ICP-MS following LiBO_2_ fusion

Analyte (MDL; ppm)	BCR-2 (ppm)			SGR-1 (ppm)		
	Measured	Certified	% Diff.	Measured	Certified	% Diff.
Sc (0.137)	32.6	33	−1.34	5.25	4.6	14.2
Y (0.003)	32.5	37	−12.1	10.3	13	−21.0
La (0.002)	24.5	25	−1.90	18.7	20	−6.4
Ce (0.002)	55.0	53	3.69	35.8	36	−0.4
Pr (0.002)	7.04	6.8	3.59	4.00	–	–
Nd (0.004)	29.6	28	5.74	14.6	16	−8.9
Sm (0.008)	6.84	6.7	2.13	2.64	2.7	−2.1
Eu (0.002)	2.04	2	2.19	0.48	0.56	−15.0
Gd (0.003)	6.85	6.8	0.70	2.13	2	6.7
Tb (0.001)	1.07	1.07	0.19	0.32	–	–
Dy (0.002)	5.54	–	–	1.65	1.9	−13.3
Ho (0.002)	1.34	1.33	0.63	0.39	–	–
Er (0.003)	3.69	–	–	1.10	1.1	0.2
Tm (0.002)	0.56	0.54	3.37	0.17	0.17	−1.5
Yb (0.002)	3.44	3.5	−1.77	1.07	0.94	13.9
Lu (0.003)	0.52	0.51	2.47	0.15	–	–

Method detection limits (MDL) are given in parentheses next to each analyte. Elements without certified values are denoted with a dash (–), as are the corresponding percent differences (% Diff.). For reference, average analytical variability was 3.4% from five sweeps during analysis.

### Mineralogical analysis

Mineralogy of the shale samples was investigated by synchrotron-based X-ray diffraction (XRD). XRD measurements were made on beamline 11-3 at the Stanford Synchrotron Radiation Lightsource (SSRL) using powdered samples. Incident X-rays ($\lambda =$ 0.9744 Å, 12,735 eV) were focused using a bent cube root I-beam Si (311) monochromator. A MAR345 area detector positioned 120 mm downstream of the sample was used to collect diffraction scans with a dwell time of 90 s. The collected images were integrated and converted into degrees 2*θ* using area diffraction machine (open source) software. The diffraction patterns were background subtracted and peak matched using Xpert Highscore Plus using a reference library obtained from the Crystallography Open Database, which was converted to synchrotron energy.

Based on previous analyses of Marcellus Shale samples, reported in Chermak and Schreiber [18], and examination of the patterns reported here, diffraction data were qualitatively partitioned to seven potential minerals (COD code in parentheses): quartz (1011097), calcite (9007867), dolomite (1200014), pyrite (5000115), illite (9013723), chlorite (9000158), and montmorillonite (9002779). The relative volume fraction of each mineral within this model assemblage was estimated for every sample by evaluation of several parameters including total diffraction peak intensity, goodness of peak fits, and contribution to the overall fitting. Additional mineral phases did not constitute significant fractions of the crystalline mineralogy and were not included. A list of the specific reference spectra used is given in the Additional file 1: Table S4.

### Data analysis

#### Rare earth element reduced dimension variables

As a convention, the REE were divided, based on atomic number, into light REE (LREE), middle REE (MREE), and heavy REE (HREE). For this study the LREE were defined to include La, Pr, Nd, and Sm; the MREE were Gd, Tb, and Dy; and the HREE were Ho, Er, Tm, Yb, and Lu. Due to their anomalous redox activity, Ce and Eu were not included in these weight-groups. Reference-normalized, interelement ratios were calculated as in Stolpe et al. [35] and Noack et al. [25], i.e. as the average of all permutations of those interelement ratios. For example, the MREE/LREE ratio for a sample was the average of $\frac{{\left[ {Gd} \right]_{N} }}{{\left[ {La} \right]_{N} }},\frac{{\left[ {Gd} \right]_{N} }}{{\left[ {Pr} \right]_{N} }},\frac{{\left[ {Gd} \right]_{N} }}{{\left[ {Nd} \right]_{N} }},\frac{{\left[ {Gd} \right]_{N} }}{{\left[ {Sm} \right]_{N} }}, {\text{etc}} .$ for all combinations of MREE and LREE where [REE]_N_ represents the reference-normalized concentration of the individual REE; the post-Archaean average shale (PAAS) of Nance and Taylor [36] was used for normalization. The anomalies of Ce and Eu (Ce* and Eu*, respectively) were calculated from PAAS-normalized concentrations (again, [REE]_N_) by Eq. 1. Alternative formulations of Eq. 1 used in the literature [37, 38] were also calculated and found to be nearly identical.

1a $$Ce^{*} = \frac{{2 \cdot \left[ {Ce} \right]_{N} }}{{\left[ {La} \right]_{N} + \left[ {Pr} \right]_{N} }}$$

1b $$Eu^{*} = \frac{{2 \cdot \left[ {Eu} \right]_{N} }}{{\left[ {Sm} \right]_{N} + \left[ {Gd} \right]_{N} }}$$

The overall degree of fractionation in a sample was defined as the sum of the absolute values of one minus each of: HREE/MREE ratios, MREE/LREE ratios, Ce anomalies, and Eu anomalies. This degree of fractionation was used to represent the overall “unevenness” or entropy of the PAAS-normalized profile. By this metric, a sample with 0 fractionation would appear flat on a plot of the PAAS-normalized concentrations for the REE while a sample with high fractionation would be highly bent, with large Ce and Eu anomalies.

#### Statistical analysis

The goals of statistical analysis were to test for differences in bulk, REE composition between core and outcrop samples as well as between northern and southern outcrops. These comparisons were made using a variety of uni- and multivariate hypothesis tests. Further, cluster and correlation analyses among XRD spectra were performed to probe differences in mineralogy, and subsequently relate those differences to bulk REE abundance and profiles. Given the small number of samples and potential non-normality of the analytes determined here, all statistical analyses were performed non-parametrically, that is, without distributional assumptions. Moreover, to control familywise error rates, Holm–Bonferroni corrections were made to all p values when utilizing multiple hypothesis tests, e.g. when comparing central tendencies of each element between core and outcrop samples. All analyses were performed using R (Version 3.1.1) and functions from the “vegan” package for multivariate analyses [39, 40]. A detailed description of these statistical methods is provided in Additional file 1 (Section: “Hypothesis tests and cluster analysis for shale comparisons”).

## Additional files

Additional file 1: Detailed descriptions of outcrop samples; ICP-MS operating parameters; statisticla validation of CRM and duplicate analyses; citations for XRD reference spectra from Crystallography Open Database; details of hypothesis testing and cluster analysis used for comparison of shale samples; details of outcrop-core statistical comparisons with associated R code; correlation analysis among the REE; correlation between reduced dimension variables (total REE and degree of fractionation) and major element composition of the outcrop samples.
