# Supplementary material for: Rare earth element geochemistry of outcrop and core samples from the Marcellus Shale
Source: Geochem Trans. 2015 Jun 26;16:6. doi: 10.1186/s12932-015-0022-4 (PMC4480892; doi:10.1186/s12932-015-0022-4)
Supplement: Additional file 1: — Detailed descriptions of outcrop samples; ICP-MS operating parameters; statisticla validation of CRM and duplicate analyses; citations for XRD reference spectra from Crystallography Open Database; details of hypothesis testing and cluster analysis used for comparison of shale samples; details of outcrop-core statistical comparisons with associated R code; correlation analysis among the REE; correlation between reduced dimension variables (total REE and degree of fractionation) and major element composition of the outcrop samples. [file 12932_2015_22_MOESM1_ESM.docx]

**Supporting information for:**

**Rare earth element geochemistry of outcrop and core samples from the Marcellus Shale**

Clinton W. Noack^1^, Jinesh Jain^2^, John Stegmeier^1,3^, J. Alexandra Hakala^4^, and Athanasios K. Karamalidis^1*^

^1^Department of Civil and Environmental Engineering, Carnegie Mellon University, Pittsburgh, Pennsylvania 15213, United States

^2^URS – Washington Division, National Energy Technology Laboratory, Pittsburgh, Pennsylvania 15236, United States

^3^Center for Environmental Implications of Nanotechnology (CEINT), United States

^4^National Energy Technology Laboratory, Pittsburgh, Pennsylvania 15236, United States

*Geochemical Transactions*

Number of pages: 20

Contains 6 Figures and 4 Tables

Clinton W. Noack

E-mail: cnoack@andrew.cmu.edu

Jinesh Jain

E-mail: [Jinesh.Jain@CONTR.NETL.DOE.GOV](mailto:Jinesh.Jain@CONTR.NETL.DOE.GOV)

John Stegmeier

E-mail: [jstegeme@andrew.cmu.edu](mailto:Jstegeme%20%3Cjstegeme@andrew.cmu.edu%3E)

J. Alexandra Hakala

E-mail: [Alexandra.Hakala@NETL.DOE.GOV](mailto:Alexandra.Hakala@NETL.DOE.GOV)

Athanasios K. Karamalidis; To whom correspondence should be addressed

Tel.: +1 412 268 1175

E-mail: [akaramal@andrew.cmu.edu](mailto:akaramal@andrew.cmu.edu)

**Sample descriptions**

Table S1. Outcrop sample names (as used in main text), locations, sampling date, descriptions, and approximate stratigraphy.

| Sample | Location | Sampling date | Lithologic description* | Stratigraphic description* |
| --- | --- | --- | --- | --- |
| Bedford, PA | 40˚ 08’ 17” N, 78˚ 35’ 01” W | 2011-09-15 | Silica-rich, non-calcareous black shale | Union Springs Member |
| Whip Gap, WV | 39˚ 16’ 10” N, 79˚ 03’ 58” W | 2011-06-29 | Non-calcareous black shale | Equivalent to basal Marcellus, presumably Union Springs Member |
| Canoga, NY (OCM) | 42˚ 51’ 20” N, 76˚ 47’ 07” W | 2011-09-15 | Sample from fresh exposure in Seneca Quarry | Oatka Creek Member |
| Canoga, NY (USM) | 42˚ 51’ 20” N, 76˚ 47’ 07” W | 2011-09-15 | Sample from fresh exposure in Seneca Quarry | Union Springs Member |
| Le Roy, NY | 42˚ 58’ 43” N, 77˚ 59’ 18” W | 2011-09-16 | Clayey shale | Oatka Creek Member of Marcellus Shale from type locality |
| Marcellus, NY | 42˚ 58’ 28” N, 76˚ 20’ 02” W | 2010-05-02 | Clayey and fissile black shale, abundant siderite concretions | Marcellus Shale type section |
| Burlington, WV (F1) | 39˚ 20’ 05” N, 78˚ 54’ 07” W | 2011-06-29 | Calcareous, organic-lean shale | Equivalent to Oatka Creek Member |
| Burlington, WV (F2) | 39˚ 00’ 11” N, 79˚ 08’ 00” W | 2011-06-29 | Shaley limestone | Equivalent to Purcell or Cherry Valley Member |
| Petersburg, WV (N) | 39˚ 00’ 41” N, 79˚ 07’ 54” W | 2011-06-29 | Calcareous black shale, fissle and organic-rich | Stratigraphically below Whip Gap sample, but part of the Marcellus and not the underlying Needmore |
| Petersburg, WV (W) | 39˚ 00’ 11” N, 79˚ 08’ 00” W | 2011-06-29 | Silty gray shale, highly friable | Stratigraphically above Whip Gap sample |

*: Lithology and stratigraphy described by sample collectors: Dr. Kathy Bruner, Dr. Richard Smosna, and Mr. Thomas Mroz.

**ICP-MS operating parameters**

Table S2. Operating conditions for ICP-MS analysis. Analysis performed on Agilent 7700x using oxygen-free argon as the carrier and dilution gas and ultra high-purity helium in the reaction cell. Conditions determined using 1000:1 diluted Agilent tuning solution.

|  | Parameter | Value |
| --- | --- | --- |
| Plasma | RF Power | 1550 W |
|  | Nebulizer pump rate | 0.10 rps |
|  | Carrier argon flow rate | 1.08 L/min |
|  | Dilution argon flow rate | 0.00 L/min |
| Lenses |  |  |
|  | Extract 1 | 0.0 V |
|  | Extract 2 | -185.0 V |
|  | Omega Bias | -110 V |
|  | Omega Lens | 8.8 V |
|  | Cell entrance | -40 V |
|  | Cell Exit | -60 V |
|  | Deflect | 1.0 V |
|  | Plate bias | -60 V |
| Octopole reaction cell |  |  |
|  | Octopole bias | -18.0 V |
|  | Octopole RF | 200 V |
|  | He flow rate | 5.0 mL/min |
|  | Energy discrimination | 5.0 V |
| Data acquisition |  |  |
|  | Replicates | 5 |
|  | Integration time | 0.3 s |
| Masses monitored |  |  |
|  | ^45^Sc, ^89^Y, ^139^La, ^140^Ce, ^141^Pr, ^145^Nd, ^147^Sm, ^151^Eu, ^157^Gd, ^159^Tb, ^163^Dy, ^165^Ho, ^167^Er, ^169^Tm, ^173^Yb, ^175^Lu | |
| Oxides and doubly charged |  | |
|  | m_2_/m_1_: 156/140, ^140^Ce^16^O^+^/^140^Ce^+^ < 0.5% | |
|  | m_2_/m_1_: 70/140, ^140^Ce^2+^/^140^Ce^+^ < 1.2% | |

**Statistical validation of CRM and duplicate analyses**

To validate our unknown sample analyses, the relative errors of certified reference material (CRM) analyses were tested if the results were balanced around zero with constant dispersion. This is analogous to validating a linear model where the error term should be normally distributed with 0 mean and fixed standard deviation, $\varepsilon\sim\mathcal{N}\left( 0,\hat{\sigma_{\varepsilon}} \right)$. In keeping with the non-parametric statistical conventions of our other analyses, this hypothesis was also tested non-parametrically. Error ($\varepsilon_{i}$) in the mass fraction of analyte *i* ($x_{i}$), presented as percent deviation from the certified value ($x_{i}^{cert}$), is calculated by Equation S1.

$\varepsilon_{i} (\%)=\frac{x_{i}-x_{i}^{cert}}{x_{i}^{cert}}\times100$ (Eqn. S1)

First, a modified test of proportions (the two-sided sign test) was used to estimate the median with confidence intervals. Acceptable results of CRM analyses should yield median errors that are not statistically significantly different than 0. The sign test was implemented using the “EnvStats” package in R, with H_0_: median equal to 0 and H_1_: true median is not equal to 0.^1, 2^ For both CRM, BCR-2 (P = 1) and SGR-1 (P=0.40), the sign test fails to reject the null hypothesis with any significant confidence, indicating that there is insufficient evidence to suggest the median errors in CRM analyses are not 0.

Next, the normality of errors was checked by fitting a normal distribution to the errors of each CRM with zero mean and standard deviation calculated directly from set of errors. The quantile-quantile plots (Q-Q plots) of these errors with fitted distribution are illustrated in Figure S1a, b. The goodness-of-fit of these normal distributions to the error data were assessed using a one-sample, two-sided Kolmogorov-Smirnov test (KS test), which uses the maximum deviation between the sample and the theoretical distribution as the test statistic. As with the sign test, the KS test fails to reject the null hypothesis that the samples come from the fitted distributions for both CRM (P_BCR-2_ = 0.33, P_SGR-1_ = 0.31).

However, visual examination (Figure S1a-d) of the sample distributions shows a strong, negative skew in BCR-2 results and a significant outlier in the SGR-1 results. Implementation of the more powerful, but parametric, Shapiro-Wilk test (SW test) for normality results in rejection of the null hypothesis for both CRM at 95% confidence. Exclusion of this outlier from the SGR-1 data (Hf, ε=42%), yields a SW test P-value of 0.51, providing confidence in the normality of the remaining analytes. Despite these findings, we have chosen to include discussion of results for Hf, understanding that there is likely significant uncertainty in the determination of this analyte. Moreover, since CRM SGR-1 is a matrix most similar to that of our unknown samples, these results (i.e. normality with mean of 0) provide confidence in our analysis.

Once more drawing from model validation, we expect that the error variance should remain constant for all observations, which, in this context, are the two CRM. Thus we tested for equal dispersion (the non-parametric equivalent of variance) between CRM results via the two-sample Ansari-Bradley test (AB test) with a H_0_: ratio of scales is 1 and H_1_: ratio of scales is not 1. With a P-value of 0.17, this test also fails to reject the null hypothesis, confirming the equal dispersion of the two CRM analyses.

Finally, to ensure the fusion method was not biasing the results, we tested for correlation between errors in analytes certified in both reference materials (n=21). As seen in Figure S1e, no correlation exists (Spearman’s ρ, P = 0.44) between the mutually certified analytes.

Taken in total, investigation of the CRM analysis errors indicates that we have reasonable confidence in our determination of unknown samples. Moreover, the rare earth elements (REE), which are the focus of this and ongoing research, exhibit some of the lowest errors among all analytes. This analysis was repeated for analytical duplicates, with similar findings (Figure S2, Table S3).

Table S3. Classical P-values of hypothesis tests for analysis of method-duplication errors in outcrop and core samples (i.e. probability of the observations given the null hypothesis). Null hypotheses (H_0_) of each test are given in parentheses. Sign test, K-S test, and S-W test are tests of the individual sample types, while A-B test and Spearman’s ρ compare errors between sample types.

| Test (H_0_) | Outcrop | Core |
| --- | --- | --- |
| Sign test  (Median = 0) | <0.01 | 0.86 |
| KS test  ($\varepsilon\sim\mathcal{N}\left( 0,\hat{\sigma_{\varepsilon}} \right)$) | <0.01 | 0.21 |
| SW test  ($\varepsilon\sim\mathcal{N}\left( \hat{\mu_{\varepsilon}},\hat{\sigma_{\varepsilon}} \right)$) | 0.76 | <0.01 |
| A-B test  (Ratio of scales = 1) | 0.36 | |
| Spearman's $\rho$  ($\rho$= 0) | 0.99 | |

Figure S1: Statistical validation of LiBO_2_ fusion method by analysis of certified reference material (CRM) errors. Errors are given as percent deviation from certified values (Eqn. S1). (a-b) Normal quantile-quantile (Q-Q) plots for CRM BCR-2 (a) and SGR-1 (b). Dashed lines correspond to normally distributed error, $\varepsilon\sim\mathcal{N}\left( 0,\hat{\sigma_{\varepsilon}} \right)$. (c-d) Frequency histograms of CRM error for BCR-2 (c; n=27) and SGR-1 (d; n=23). (e) Error biplot for elements with certified values in both CRM (n=21).

Figure S2: Statistical validation of LiBO_2_ fusion method by analysis method duplicate errors. Errors are given as percent deviation from certified values (Eqn. S1). (a-b) Normal quantile-quantile (Q-Q) plots for outcrop duplicates (a) and core duplicates (b). Dashed lines correspond to normally distributed error, $\varepsilon\sim\mathcal{N}\left( 0,\hat{\sigma_{\varepsilon}} \right)$. (c-d) Frequency histograms of duplicate error for outcrop (c; n=30) and core (d; n=30) (e) Paried error biplot for analytes in duplicates (n=30).

**XRD reference spectra**

Table S4. Crystallography Open Database (COD) codes and references for model compounds fit to XRD spectra obtained for samples in this study.

**Hypothesis tests and cluster analysis for shale comparisons**

Univariate statistical tests were used to compare the REE distributions between core and outcrop samples as well as between northern and southern outcrops. Individual elements were compared between sample types to assess differences in central tendency (Wilcoxon rank-sum test) and dispersion (Ansari-Bradley test). Both tests were evaluated as two-sided tests (i.e. H_0_: no difference in median/dispersion) with resulting P-values corrected for multiple comparisons using the Holm-Bonferroni method. P-value adjustments are particularly important within this dataset given the small sample size and numerous analytes. Details of these procedures as they pertain to the outcrop versus core comparison are detailed, including R source code necessary for reproduction, in the SI section “Outcrop-core statistical comparison”.

However, given the multivariate nature of this data set, it was also useful to utilize a multivariate test. Here a permuted, multivariate analysis of variance test (PERMANOVA) was used.^3^ This test partitions distance matrices among sources of variance (i.e. “core” or “outcrop”) and uses a permutation test to determine significance. Intersample distances for the PERMANOVA test were calculated using the Bray-Curtis metric,^4^ which normalizes differences in a variable between two samples to the sum of that variable in those samples, creating a metric robust to differences in variable scales. To restate, the Bray-Curtis metric will not bias the distance between samples to the variables with the highest values where a Euclidean distance would. For example, the LREE are typically highly concentrated relative to the HREE (by an order of magnitude or more); a Euclidean distance would be biased towards differences in the LREE while the Bray-Curtis would not.

Cluster analysis was used to compare between individual samples on the basis of XRD patterns. Cluster analysis for the XRD pattern was performed by first calculating the intersample distance as one minus the Spearman’s $\rho$ correlation between the relative intensity (i.e. normalized to the sample maxima) of diffraction spectra over the $2\theta$ interval of 10˚ – 45˚. A similar approach was used by Long et al.^5^ to determine the distribution of phases in ternary metallic alloys. Clusters were determined using an unweighted, average-distance algorithm. The results of this cluster analysis allows for more quantitative, and visually compelling, comparison among spectra. The PERMANOVA test was also used to assess group differences (i.e. between core and outcrop) in mineralogies, also making use of the correlation-based distance (again, one minus the Spearman’s $\rho$ statistic).

Relationships between mineralogy and REE profiles/abundance were investigated by correlation and regression analysis. The Mantel test^6^ was used to examine correlations between distance matrices. REE profiles were compared to the XRD spectra (as before, over the $2\theta$ interval of 10˚ – 45˚) by taking the Bray-Curtis distance of the REE data and testing for correlation with the Spearman’s $\rho$ distance of the XRD spectra. In an attempt to hypothesize the mineralogy of the REE, both abundance and fractionation were compared between samples based on major mineralogy. That is, a Wilcoxon rank-sum test was used to compare the median total REE content (or median fractionation) in samples which had a given mineral as a major phase with those that did not. This analysis was repeated for each of the six model minerals fit to the XRD data. Use of the Wilcoxon test also allows for calculation of the Hodges-Lehmann estimator (HL) of location shift (i.e. the approximate difference in the group medians) along with a confidence interval on this estimator.

**Outcrop-core statistical comparison**

Statistical comparison between core and outcrop samples was performed with complementary parametric and non-parametric tests of central tendency (two-sample t-tests and Wilcoxon tests) and homogeneity of variance/dispersion (Bartlett tests and Ansari-Bradley tests). Here is a summary of that analysis, performed in R version 3.1.1 (2014-07-10). This analysis utilizes statistical routines built into base R, but also makes use of extended packages: plyr, dplyr, and tidyr.^7-9^ Functions from these namespaces are denoted as package_name::function_name, e.g. dplyr::mutate.

library(plyr, warn.conflicts = F)
library(dplyr, warn.conflicts = F)
library(tidyr, warn.conflicts = F)

The data, provided in Table 2 of the main text, are loaded and samples are assigned to core or outcrop groups based on their names. Samples generically labeled "C-*N*" represent a core at depth *N* (ft bgs), however sample "1-DGLS" is a core that does not adhere to that convention. All other samples are outcrops. Duplicates are not removed from this analysis.

REE <- read.table(file='Raw Data/ShaleREE_LMB_final.txt',
 sep='\t',header=T)
# REE concentrations in ppm.
dplyr::tbl_df(REE)

## Source: local data frame [18 x 15]
##
## Sample La Ce Pr Nd Sm Eu Gd
## 1 Bedford, PA 15.30 30.85 4.142 16.215 3.743 0.8077 4.495
## 2 Canoga, NY (OCM; D1) 26.45 44.16 6.538 26.061 5.989 1.3126 6.486
## 3 1-DGLS 18.97 34.44 4.889 19.977 4.882 1.0685 5.163
## 4 Petersburg, WV (N) 32.16 65.38 7.901 31.760 6.228 1.2671 5.885
## 5 C-7789 28.69 61.22 7.140 26.593 4.988 1.1334 4.461
## 6 Whip Gap, WV 12.72 21.14 2.749 9.649 1.879 0.4078 1.828
## 7 Burlington, WV (F1) 44.69 96.95 11.020 42.150 7.831 1.5985 6.377
## 8 Canoga, NY (USM) 38.35 75.64 9.170 33.397 6.546 1.4760 6.625
## 9 C-7838 38.64 75.87 9.077 34.794 6.819 1.4616 6.252
## 10 Petersburg, WV (W) 45.48 96.52 11.188 40.274 7.864 1.6239 6.619
## 11 C-7907 37.09 52.97 5.898 19.332 3.215 0.6440 2.901
## 12 C-7801 (D1) 39.60 81.60 10.026 37.499 7.947 1.5293 7.013
## 13 C-7801 (D2) 39.50 81.86 9.877 38.428 8.138 1.5370 7.052
## 14 Le Roy, NY 35.45 73.37 9.264 36.235 7.992 1.7701 7.809
## 15 Marcellus, NY 42.54 88.67 10.340 39.286 7.803 1.6423 7.186
## 16 Burlington, WV (F2) 18.24 32.46 4.282 18.362 3.921 1.1751 4.703
## 17 C-7813 40.38 79.50 9.522 34.849 6.824 1.3274 5.509
## 18 Canoga, NY (OCM; D2) 26.53 42.48 6.439 26.090 5.714 1.2182 6.960
## Variables not shown: Tb (dbl), Dy (dbl), Ho (dbl), Er (dbl), Tm (dbl), Yb
## (dbl), Lu (dbl)

names <- as.character(REE$Sample)
cores <- c(names[ grep("1-DGLS",names)], names[ grep("C.7",names)])
core_logical <- names %in% cores

REE <- REE %>%
 dplyr::select(-Sample) %>%
 dplyr::mutate(type = factor(ifelse(core_logical, 'Core','Outcrop')))

To analyze element-by-element, the data are gathered using the element as a qualitative key. The resulting data is divided by element and the p-values of two-sided tests are returned for each subset. Results show that, even before correction for multiple comparisons, there are no significant results at any conventional P-value (e.g. $\alpha=0.05$). Conclusions from parametric tests are equivalent with or without log-transformation of the concentrations.

REE_melt <- tidyr::gather(REE, element, concentration, -type)

p.vals <- plyr::ddply(REE_melt, .(element), function(df){
 ## Dispersion/variance tests
 # Non-parametric
 ab <- ansari.test(concentration ~ type, data = df)$p.value
 # Parametric
 bt <- bartlett.test(concentration ~ type, data = df)$p.value

 ## Central tendency tests
 # Non-parametric
 wt <- wilcox.test(concentration ~ type, data = df)$p.value
 # Parametric
 t <- t.test(concentration ~ type, data = df)$p.value

 data.frame(Bartlett = bt, Ansari = ab, Students.t = t, Wilcox = wt)
})

dplyr::tbl_df(p.vals)

## Source: local data frame [14 x 5]
##
## element Bartlett Ansari Students.t Wilcox
## 1 La 0.3255 0.2436 0.4067 0.4789
## 2 Ce 0.2823 0.3253 0.5800 0.5962
## 3 Pr 0.3764 0.3253 0.6708 0.8601
## 4 Nd 0.4788 0.3253 0.7976 0.8601
## 5 Sm 0.7718 0.6572 0.8633 0.7914
## 6 Eu 0.5637 0.5334 0.7455 0.5360
## 7 Gd 0.7521 0.9295 0.5794 0.4789
## 8 Tb 0.7951 0.7900 0.7391 0.7242
## 9 Dy 0.7322 0.7900 0.6971 0.5962
## 10 Ho 0.7335 0.7900 0.8331 0.9298
## 11 Er 0.6543 0.6572 0.9829 0.7914
## 12 Tm 0.6577 0.7900 0.8426 0.7242
## 13 Yb 0.4667 0.9295 0.7907 0.6590
## 14 Lu 0.3950 0.6572 0.7092 0.6590

# Are any P-values less than 0.05?
p.vals %>% tidyr::gather(test, p.val, -element) %>%

dplyr::summarize(any(p.val < 0.05))

## any(p.val < 0.05)
## 1 FALSE

Correction of these P-values for multiple comparisons using the Holm-Bonferroni method further diminishes any statistical significance of these comparisons.

# Correct P-values for each test across elements being compared
p.vals.adj <- p.vals %>%
 tidyr::gather(test, p.val, -element) %>%
 dplyr::group_by(test) %>%
 dplyr::mutate(p.val = p.adjust(p.val, method = 'holm')) %>%
 dplyr::ungroup() %>%
 tidyr::spread(key = test, value = p.val)

dplyr::tbl_df(p.vals.adj)

## Source: local data frame [14 x 5]
##
## element Bartlett Ansari Students.t Wilcox
## 1 La 1 1 1 1
## 2 Ce 1 1 1 1
## 3 Pr 1 1 1 1
## 4 Nd 1 1 1 1
## 5 Sm 1 1 1 1
## 6 Eu 1 1 1 1
## 7 Gd 1 1 1 1
## 8 Tb 1 1 1 1
## 9 Dy 1 1 1 1
## 10 Ho 1 1 1 1
## 11 Er 1 1 1 1
## 12 Tm 1 1 1 1
## 13 Yb 1 1 1 1
## 14 Lu 1 1 1 1

Using power analysis, we can determine how many samples of each type would be necessary to detect a statistically significant ($\alpha=0.05$) result for typical powers, i.e. $(1-\beta)\in\{0.8,0.9\}$, given the differences observed in the current dataset. This utilizes code written in the pwr and effsize packages. From this analysis, it is shown that somewhere between ~100 – 200,000 samples would be needed in each group to flag these differences as “statistically significant”, before correction for multiple comparisons. Conservatively (i.e. using the Bonferroni adjustment for *k* comparisons, $\alpha_{Bonf}=\frac{\alpha}{k}$), statistically significant results for corrected P-values would require just less than twice as many samples (analysis not shown).

library(effsize, warn.conflicts = F)

## Warning: package 'effsize' was built under R version 3.1.2

library(pwr, warn.conflicts = F)

## Warning: package 'pwr' was built under R version 3.1.3

eff_size <- REE_melt %>%
 plyr::ddply(.(element), function(df){
 effsize::cohen.d(df$concentration, df$type)$estimate
 }
 )

# Determine practical significance of Cohen's d for observed differences
eff_size <- eff_size %>%
 mutate(Core = abs(Core),
 practical = cut(Core,
 breaks = c(0,0.2,0.5,0.8,Inf),
 labels = c('negligible','small','medium','large')))

dplyr::tbl_df(eff_size)

## Source: local data frame [14 x 3]
##
## element Core practical
## 1 La 0.37787 small
## 2 Ce 0.24852 small
## 3 Pr 0.19363 negligible
## 4 Nd 0.11843 negligible
## 5 Sm 0.08262 negligible
## 6 Eu 0.15180 negligible
## 7 Gd 0.26664 small
## 8 Tb 0.16049 negligible
## 9 Dy 0.18623 negligible
## 10 Ho 0.10065 negligible
## 11 Er 0.01014 negligible
## 12 Tm 0.09391 negligible
## 13 Yb 0.12240 negligible
## 14 Lu 0.17026 negligible

# Determine samples needed for statistical significance of observed effect size
samps_needed <- eff_size %>%
 ddply(.(element), function(df){
 power_0.80 = pwr::pwr.t.test(d = df$Core, sig.level = 0.05, power = 0.8)$n %>%
 round()
 power_0.90 = pwr::pwr.t.test(d = df$Core, sig.level = 0.05, power = 0.9)$n %>%
 round()
 data.frame(power_0.80, power_0.90)
 })

dplyr::tbl_df(samps_needed)

## Source: local data frame [14 x 3]
##
## element power_0.80 power_0.90
## 1 La 111 148
## 2 Ce 255 341
## 3 Pr 420 561
## 4 Nd 1120 1499
## 5 Sm 2301 3080
## 6 Eu 682 913
## 7 Gd 222 297
## 8 Tb 610 817
## 9 Dy 454 607
## 10 Ho 1551 2076
## 11 Er 152599 204287
## 12 Tm 1781 2384
## 13 Yb 1049 1404
## 14 Lu 542 726

Figure S3. Relationship between interelement correlation (Spearman’s $\rho$) and difference in atomic number (top) and atomic radii^10^ (bottom) in Marcellus Shale samples. The critical value ($\alpha=0.05$) for a positive correlation between elements with 18 observations is noted with the dashed line.

Figure S5. Scatter plots showing total REE mass fraction as a function of major element mass fraction. Data from Dilmore et al.^11^ are plotted along with fitted, linear predictors and 95% prediction intervals. For P and Mn, correlation is not significant after removal of outliers.

Figure S6. Scatter plots showing degree of REE profile fractionation as a function of major element mass fraction. Data from Dilmore et al.^11^ are plotted along with fitted, linear predictors and 95% prediction intervals. For P and Mn, correlation is not significant after removal of outliers.

**References**

1. Millard, S. P.; Neerchal, N. K.; Dixon, P., *Environmental Statistics with R*. CRC: 2012.

2. R Core Team *R: A Language and Environment for Statistical Computing*, 3.0.3; R Foundation for Statistical Computing: Vienna, Austria, 2014.

3. Anderson, M. J., A new method for non-parametric multivariate analysis of variance. *Austral Ecol.* **2001,** *26*, (1), 32-46.

4. Bray, J. R.; Curtis, J. T., An ordination of the upland forest communities of southern Wisconsin. *Ecol. Monogr.* **1957,** *27*, (4), 325-349.

5. Long, C. J.; Hattrick-Simpers, J.; Murakami, M.; Srivastava, R. C.; Takeuchi, I.; Karen, V. L.; Li, X., Rapid structural mapping of ternary metallic alloy systems using the combinatorial approach and cluster analysis. *Rev. Sci. Instrum.* **2007,** *78*, (7), -.

6. Mantel, N., The detection of disease clustering and a generalized regression approach. *Cancer Res.* **1967,** *27*, (2 Part 1), 209-220.

7. Wickham, H.; Francois, R. *dplyr: A Grammar of Data Manipulation*, R package version 0.3.0.3; CRAN, 2014.

8. Wickham, H. *tidyr: Easily tidy data with spread and gather functions*, R package version 0.1; CRAN, 2014.

9. Wickham, H., The split-apply-combine strategy for data analysis. *Journal of Statistical Software* **2011,** *40*, (1), 1-29.

10. Shannon, R., Revised effective ionic radii and systematic studies of interatomic distances in halides and chalcogenides. *Acta Crystallographica Section A* **1976,** *32*, (5), 751-767.

11. Dilmore, R.; Bruner, K.; Wyatt, C.; Romanov, V.; Hedges, S.; Crandall, D.; Disenhof, C.; Jain, J. C.; Lopano, C.; Aminian, K.; Zamirian, M.; Mashayekhi, A.; Mroz, T.; Soeder, D. J. *2012 ICMI Carbon Storage in Depleted Shale: Experimental Program Summary Report*; U.S. Department of Energy National Energy Technology Laboratory: 2012.
